# Why Human Subjects Research Protection Is Important

**DOI:** 10.31486/toj.20.5012

**Published:** 2020

**Authors:** Michael G. White

**Affiliations:** Department of Pediatric Cardiology, Ochsner Clinic Foundation, New Orleans, LA and The University of Queensland Faculty of Medicine, Ochsner Clinical School, New Orleans, LA

**Keywords:** *Ethics committees–research*, *ethics–research*, *research subjects*

## Abstract

**Background:** Institutional review boards (IRBs), duly constituted under the Office of Human Research Protection, have the federally mandated responsibility of reviewing research involving human subjects to ensure that a proposed protocol meets the appropriate ethical guidelines before subjects may be enrolled in any study. The road leading to the current regulations and ethical considerations has been long and checkered.

**Methods:** This paper reviews the history of human subjects participating in research, including examples of egregious events, and the ethical analyses that precipitated the evolution of the mandated protections afforded participants in research under current federal regulations.

**Results:** Key documents—from the Nuremberg Code in 1947 to the Belmont Report in 1978 to *Moral Science: Protecting Participants in Human Subjects Research* in 2011—that have informed the ethics debate regarding human subjects protection in research activities are presented in light of their historic significance, highlighting the complexity of the issues surrounding protection of human subjects in research.

**Conclusion:** The examples from history and the scarcity of contemporary examples demonstrate that the regulations for the protection of humans participating in research have evolved in a way that minimizes the probability that subjects will be harmed when they choose to participate in research. The examples also reinforce the importance of individual responsibility. Failure of IRBs to provide appropriate review and oversight can lead to severe consequences, as can abrogation by the investigator to place the well-being of the subjects as the primary responsibility in any research protocol. Understanding how we arrived at the current approach and some of the failures that directed this course can support efforts to continually reevaluate and improve the safety of subjects who are willing to participate in research activities.

## INTRODUCTION

Participation of human subjects in research presents a challenging ethical dilemma. A research subject may be asked to participate in a study of no benefit and no substantial risk or in a study with the potential for significant benefit but also significant risk. In placebo-controlled studies, subjects may be exposed to significant risk for no benefit to the individual. These variants are confounded by treatment protocols—most commonly encountered in oncology trials—that compare the effect of an investigational arm to the standard of care, further blurring the distinction between research and medical treatment.

Institutional review boards (IRBs) have the federally mandated responsibility to review research involving human subjects to ensure that a proposed protocol meets the appropriate ethical guidelines before subjects may be enrolled in the study. The road leading to the current regulations and ethical considerations has been long and checkered. The system that has evolved minimizes the risks for unethical behavior and serious adverse events but is not infallible. Understanding how we have arrived at the current approach and analyzing some of the ethical lapses that directed this course support efforts to continually reevaluate the regulations in order to improve the safety of subjects who are willing to participate in research activities.

## EVOLUTION OF HUMAN SUBJECTS PROTECTION

Our current approach to human subjects protection has evolved with efforts to understand questionable ethical behavior in research over the course of several hundred years. One might suggest that the jester conscripted to sample the king's food to ensure that it was safe to eat presaged the use of vulnerable populations as subjects for research, but the evolution of the management of smallpox is perhaps a more applicable early perspective on research in humans. Three centuries ago, reports of good outcomes following variolation—inhalation of the scabs from persons infected with smallpox—were circulating in Asia. In 1717, Lady Mary Wortley Montagu, the wife of the British ambassador to Turkey, became an advocate of variolation after learning about it in Constantinople. In 1721, after she returned to England, Lady Montagu and the Princess of Wales urged variolation of “several prisoners and abandoned children” by having smallpox scabs inserted under their skin. Several months later, the children and prisoners were deliberately exposed to smallpox. When none contracted the disease, the procedure was deemed safe, and members of the royal family were treated according to this new protocol.^1^

Later that same century, Edward Jenner developed inoculation with a vaccine. Many of his contemporaries had noted that milkmaids who had contracted cowpox seemed immune to the much more lethal smallpox. In May 1796, Jenner isolated material from the cowpox lesions on the milkmaid Sarah Nelms and inoculated 8-year-old James Phipps who developed fever and malaise about 9 days after the inoculation. Some accounts report that Phipps was the son of Jenner's gardener. A few months later, Jenner deliberately inoculated Phipps with material from fresh smallpox lesions, and the child remained healthy. The adoption of this process was not immediate but slowly spread and is widely cited as the first scientific approach proving vaccination.^2^

This early use of children and prisoners portends a long history of selecting what are now considered vulnerable populations to be the subjects of research. Participation was commonly without consent, with no knowledge of their participation, and with no explanation of the research. Information was withheld from those selected to participate in research activities perceived as dangerous to more *acceptable* members of society, and the therapies developed were generalized only if they were proven relatively safe and effective in what are now recognized as vulnerable populations.

Numerous instances of research experiments in subsequent years exposed vulnerable subjects to risk, including a pivotal research disaster in Germany just before World War II that led to regulations for human subjects participation in research projects.

### The Reich Circular of 1931

As reported by Sir Graham Wilson in the book *The Hazards of Immunization*, “Between 10 December 1929 and 30 April 1930, 251 of 412 infants born in the old Hanseatic town of Lubeck received three doses of BCG [bacillus Calmette-Guerin] vaccine by the mouth during the first ten days of life. Of these 251, 72 died of tuberculosis, most of them in two to five months and all but one before the end of the first year. In addition, 135 suffered from clinical tuberculosis but eventually recovered; and 44 became tuberculin-positive but remained well.”^3^

Bonah and Menut describe how Albert Calmette was able to establish the BCG vaccine as a nonexperimental “prophylactic treatment” against tuberculosis.^4^ By definition, a medical experiment, as opposed to any other medical action, has definite ethical implications and consequences. Even though the BCG vaccine was in experimental stages, Calmette convinced a court that the vaccine was a “post-experimental, routine medical treatment.” By avoiding the definition of an experiment, Calmette did not have to inform the children's parents about the risks of the vaccine. As a result of this tragedy, Dr Julius Moses, a critic of unethical human experimentation who referred to “experimental mania,” drafted guidelines for human experimentation. After debate in parliament and the press, the guidelines were published and became official in 1931. The guidelines applied to everyone in Germany.^5,6^

**Figure 1.**
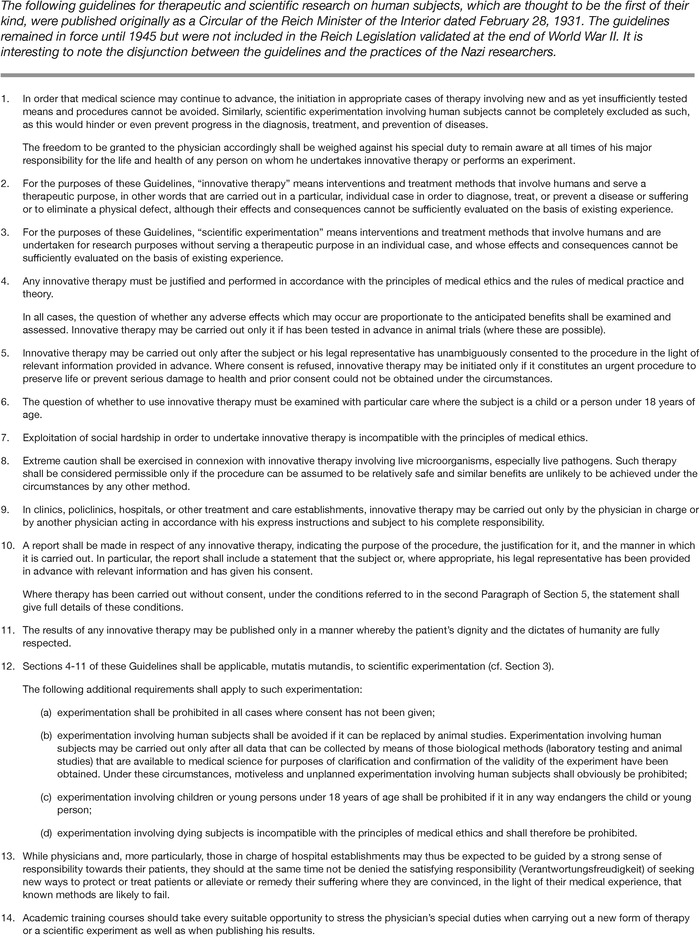
**The Reich Circular, 1931^6^**

These rules for research in human subjects were issued as the Reich Circular of 1931 (Figure 1). The document is quite informative for its contrast with later events in Germany and worth reviewing for correlation with ethical concepts now well accepted in ethical thinking. It is worth noting that these guidelines emphasize special responsibilities for utilization of “innovative therapy,” suggesting a similar level of responsibility for these procedures as for research.

### World War II and the Nuremberg Code

Despite the ethical ideals espoused in the Reich Circular, the travesty of the Holocaust followed shortly afterward, leading to war criminal trials after the surrender of Germany ended World War II in Europe.

The Nuremberg trials that began in 1945 and concluded in 1947 were held in response to the atrocities Germany committed during the war. The so-called Doctors’ Trial represents a major turning point in human research protection. Twenty-three physicians were indicted, accused of crimes against humanity by conducting criminal scientific and medical experiments on concentration camp prisoners. Sixteen defendants were found guilty.^7^

**Figure 2.**
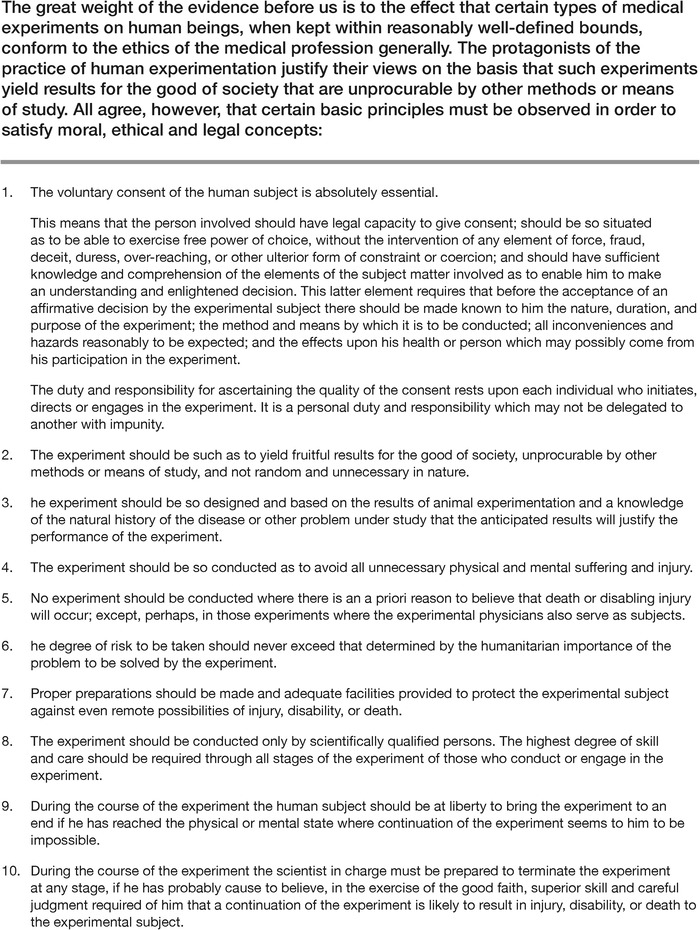
**The Nuremberg Code, 1947^10^**

Several German doctors had argued that no international law or informal statement differentiated between legal and illegal human experimentation, despite the aforementioned Reich Circular. Two US doctors who worked with the prosecution during the trial, Andrew Ivy and Leo Alexander, objected to this argument. On April 17, 1947, Dr Alexander submitted a memorandum to the United States Counsel for War Crimes outlining 6 points defining legitimate medical research. The trial verdict reiterated almost all of these points in a section entitled Permissible Medical Experiments and expanded the original 6 points into 10. These 10 points became known as the Nuremberg Code (Figure 2).^8^

Similar atrocities were carried out on Chinese citizens in Japanese camps; of particular note are the biological warfare experiments at Unit 731 in the Pacific theater that were obscured by agreements made during the surrender of Japan and with the complicity of the United States.^9^ The details of these atrocities remained classified until they were acknowledged by Congress in the Japanese Imperial Government Disclosure Act of 2000 (Pub L No. 106-567, Title VIII of the Intelligence Authorization Act of 2000) that called for declassification and release of records related to Japanese war crimes during World War II.^10^

### Declaration of Helsinki

The tenets of the Nuremberg Code, while guiding the future for human research protection, represent a military code of conduct with no standing in civil international or US law. By absolutely requiring the voluntary consent of the individual, the Nuremberg Code notably does not address the needs of children or other special populations unable to provide consent. The Nuremberg Code inspired the World Medical Association (WMA)—an international association currently comprised of 114 national medical associations, including the American Medical Association—to propose a similar code of conduct for participating members by publishing the Declaration of Helsinki in 1964. This document reiterates the provisions of the Nuremberg Code and expands the provisions to allow for the participation of children and other potentially compromised subjects in research. The Declaration of Helsinki serves as a guideline for ethical research and has been amended 7 times, most recently at the WMA General Assembly in October 2013, to reflect contemporary ethical issues as they have evolved since the initial statement in 1964.^11^

### Ethics Violations in the United States

Meanwhile, research continued in the United States with particular concerns attached to research involving vulnerable populations, exemplified by numerous studies involving institutionalized children and studies that breached ethically sound research practices. Henry Beecher, a well-recognized physician at Massachusetts General Hospital, surveyed the contemporary literature to identify ethical concerns and organized lectures around his observations. These lectures eventually culminated in a special article published in the *New England Journal of Medicine* in 1966.^12^

In “Ethics and Clinical Research,” Beecher reported that he had reviewed 100 consecutive articles published in 1964 “in an excellent journal,” and after culling his list to address the editor's request, selected 12 articles that demonstrated serious ethical concerns. The purpose of Beecher's article was to demonstrate the widespread lapse in ethical issues in medical research and to encourage reform in the ethical approach to human subjects research that inspired Congress to reconsider legislative reforms for human subjects protection.

An article by Jean Heller that appeared in the *Washington Star* on July 25, 1972 placed an exclamation point in the history of human research ethics.^13^ Heller reported on a long-term study sponsored by the US Public Health Service on the effect of syphilis if left untreated in poor rural African American subjects. Officially known as the “Tuskegee Study of Untreated Syphilis in the Negro Male,” the study enrolled 399 subjects with syphilis and 201 uninfected controls from the African American community surrounding Tuskegee, AL for “treatment of bad blood.” In exchange for taking part in the study, the men received free medical examinations, free meals, and burial insurance but were not given the benefit of providing informed consent. No treatment was provided; the research plan was to follow the subjects to establish a natural history for the disease if left untreated. Although originally projected to last 6 months, the study continued for 40 years.^14^

Treatments available at the onset of the trial in 1932, even if provided, were not very effective and would have been heavy metals, involving at least 30 months of treatment, a 30% cure rate, and significant toxicity. By 1945, penicillin had been proven to be an effective therapy for syphilis with few side effects. Once penicillin was established as effective, the US Public Health Service set up centers for treatment but determined that the data from the Tuskegee experiments were too important to abandon and decided that the study should be continued with no treatment provided to the participants. Similar determinations were made in subsequent years, with the last review occurring as recently as 1969.^14^

While medical research such as the Tuskegee study garnered most of the attention for ethical lapses, other areas of research involving human subjects also raised concerns. The Milgram experiments carried out in the early 1960s at Yale University are a lightning rod for discussion of ethical issues in human subjects research in social sciences.^15^ Intrigued by the Nuremberg trials defendants’ argument that they were simply following orders, Stanley Milgram set out to determine if the German defendants were particularly obedient to authority figures compared to other members of society. Milgram recruited subjects for an experiment in learning via newspaper ads. The male research subjects were assigned to act as a teacher asking questions of a learner (a confederate of Milgram) who was attached to electrodes. The teachers were instructed to increase the severity of electrical shocks if the learner answered the questions incorrectly. Shocks were labeled from 15v to 450v, with 15v indicated as mild, 300v as severe, and 450v as XXX. Many of the teacher subjects eventually shocked the learner at 450v and exhibited increasing signs of distress as the shocks they delivered increased in perceived severity.^15^ These experiments evoked significant concern among those in social sciences in regard to the questionable ethics of the deception used, as well as the potential for long-term psychological harm that might be incurred by unwitting participants.

### Federal Policy for Protection of Human Subjects and the National Research Act

The public outcry over the Tuskegee study, other reports of ethical lapses in both medical and social research, and the alarm in the medical community raised by Dr Beecher's article in the *New England Journal of Medicine* led Congress to action. On May 30, 1974, the US Department of Health, Education, and Welfare (DHEW), responsible for oversight of the National Institutes of Health, replaced previous policies with comprehensive regulations governing the protection of human subjects (45 CFR §46).^16^ One month later in July 1974, Congress passed the National Research Service Award Act of 1974 (Pub L No. 93-348).^17^ Title II of the act, Protection of Human Subjects of Biomedical and Behavioral Research, created the National Commission for the Protection of Human Subjects in Biomedical and Behavioral Research. Along with being assigned several other tasks, the National Commission was directed to make recommendations to the DHEW secretary about the ethical principles that should underlie human subjects research.^18^

### The Belmont Report

The National Commission issued several reports in response to the directives. The most notable among a collection of important documents is the Belmont Report, named after the Smithsonian conference center where the group convened, that was issued in 1978.^19^ This document, widely regarded as the landmark analysis of ethics in human subjects research, serves as the foundation for discussion of ethical concerns in research ethics involving human subjects, as well as the source of federal regulations for research established by the Office for Human Research Protections (OHRP).

The Belmont Report is divided into three sections. The first section briefly states the National Commission's recognition that even as the report was being written, the distinction between medical practice and research was blurred. The report defines medical practice as “interventions that are designed solely to enhance the well-being of an individual patient or client and that have a reasonable expectation for success. Research, on the other hand, is defined as “an activity designed to test an hypothesis, permit conclusions to be drawn, and thereby to develop or contribute to generalizable knowledge (expressed, for example, in theories, principles, and statements of relationships).” This section further expounds on the conflation between the use of the terms experimental and research. When used in reference to a procedure or treatment that significantly deviates from typical (ie, a treatment that is “new, different or untested”), the report notes that an “experimental” treatment is not necessarily research. Although they excluded “experimental” treatment from research and the applicable anticipated regulations, the National Commission strongly recommended that such treatments should eventually be incorporated into formal research protocols “to determine if they are safe and effective.” The first section of the Belmont Report concludes with the recognition that practice and research may go hand in hand: “the general rule is that if there is any element of research in an activity, that activity should undergo review for the protection of human subjects.”^19^ This language is reminiscent of the Reich Circular recommendation regarding “innovative therapy.”

The second section is the heart of the report and defines three principles that should guide the discourse surrounding any ethical concerns related to research in human subjects: respect for persons, beneficence, and justice. The principle of nonmaleficence, now commonly accepted as one of the four principles of biomedical ethics, was notably absent.

- **Respect for persons**: The principle of respect for persons requires that “individuals should be treated as autonomous agents,” and those with “diminished autonomy are entitled to protection.” These concepts inform “two separate moral requirements: the requirement to acknowledge autonomy and the requirement to protect those with diminished autonomy.” The National Commission defines the elements that would be necessary to qualify as an autonomous individual and explores circumstances that would define those who should be considered to be of diminished autonomy and thus deserving of protection.
- **Beneficence**: The principle of beneficence as defined by the National Commission encompasses the concept of do no harm included in the Hippocratic Oath and notes that the term is commonly thought “to cover acts of kindness or charity that go beyond strict obligation.” The National Commission proposes two general rules that inform beneficence as an obligation: “(1) do not harm and (2) maximize possible benefits and minimize possible harms.” The implications of these duties within the context of both individual investigators and society at large are examined.
- **Justice**: The principle of justice is posed as the following question: “Who ought to receive the benefits of research and bear its burdens?” This principle is broad in potential implications and can be summarized as evaluating the appropriate distribution of the risks and burdens of research among individuals, groups, or even situations in which inherent inequalities may need to be considered to reach an ethically informed decision. The National Commission proposes the following framework for beginning these discussions: “(1) to each person an equal share, (2) to each person according to individual need, (3) to each person according to individual effort, (4) to each person according to societal contribution, and (5) to each person according to merit.” The discussion of justice continues with the historic context for including the principle of justice and how lapses in justice (ie, the Tuskegee study) were the primary impetus for the formation of the National Commission.

The final section of the Belmont Report addresses the application of these principles and the implications of their requirements when considering three important elements of research involving human subjects: informed consent, assessment of risks and benefits, and selection of subjects for research.

*Informed consent.* The consent process has three components: information, comprehension, and voluntariness. Reaching agreement on an appropriate standard for evaluating the quality of information that should be provided to potential participants about a proposed research project is difficult and eventually ends with the suggestion that the standard of “the reasonable volunteer” might best fulfill the requirements of respect for persons, beneficence, and justice. A caveat is provided, citing the problem posed by research where “informing subjects of some pertinent aspect of the research is likely to impair the validity of the research,” a key area of ethical concern (lack of disclosure) raised by the Milgram study discussed previously. The National Commission proposes that such studies may only be appropriate if “(1) incomplete disclosure is truly necessary to accomplish the goals of the research, (2) there are no undisclosed risks to subjects that are more than minimal, and (3) there is an adequate plan for debriefing subjects, when appropriate, and for dissemination of research results to them,” further noting that “Care should be taken to distinguish cases in which disclosure would destroy or invalidate the research from cases in which disclosure would simply inconvenience the investigator.”^19^

Regarding the component of comprehension, the Belmont Report states, “The manner and context in which information is conveyed is as important as the information itself.” The level of comprehension is also important within the context of the individual's ability to understand the information, with emphasis that the obligation for ensuring subject understanding increases in importance relative to the level of risk posed by participation in the study. The National Commission suggests that some level of questioning the subject to ensure comprehension is appropriate and even suggests that written responses to questions may be appropriate if risks are exceptionally high.^19^ If participation of subjects with compromised abilities is anticipated, researchers must be particularly diligent in evaluating the level of comprehension by the subject's proxy and ensure that the proxy is indeed capable of representing the best interests of the subject. The report even suggests that the proxy might need to be present or available during the research interventions to withdraw the subject from the study if the proxy perceives that withdrawal may be in the subject's best interest.

Voluntariness is a concept consistently emphasized in the Reich Circular, the Nuremberg Code, the Declaration of Helsinki, and the Belmont Report. Although voluntariness may appear to be self-evident, it may be the most difficult concept to address. The Belmont Report emphasizes that the subject must be “free of coercion and undue influence.” Coercion is specifically defined as “an overt threat of harm” and in most circumstances is relatively easy to evaluate. However, arguments can be made about what defines “undue influence.” Discussions about appropriate levels of compensation for participation are common, particularly when studies involve financial or other considerations made to possibly financially compromised subjects. The Belmont Report specifically notes, “inducements that would ordinarily be acceptable may become undue influences if the subject is especially vulnerable.” Other concerns related to undue influence involve social standing, employment, or other circumstances that may be difficult to assess but are worthy of consideration for individual subjects.

*Assessment of risks and benefits.* The National Commission notes that a favorable risk/benefit assessment is associated with the principle of beneficence. This definition is particularly appropriate in that the National Commission's interpretation of beneficence includes the duty of nonmaleficence. The Belmont Report examines the meaning of risk and benefit in the setting of potential types of harm that may be experienced by individual subjects, the families of the individual subjects, society at large, or special groups of subjects in society. Benefits are also discussed in relation to the individual and society at large. In summarizing the risks and benefits of research, the Belmont Report states

…assessment of the justifiability of research should reflect at least the following considerations:

Brutal or inhumane treatment of human subjects is never morally justified.

Risks should be reduced to those necessary to achieve the research objective. It should be determined whether it is in fact necessary to use human subjects at all. Risk can perhaps never be entirely eliminated, but it can often be reduced by careful attention to alternative procedures.

When research involves significant risk of serious impairment, review committees should be extraordinarily insistent on the justification of the risk (looking usually to the likelihood of benefit to the subject—or, in some rare cases, to the manifest voluntariness of the participation).

When vulnerable populations are involved in research, the appropriateness of involving them should itself be demonstrated. A number of variables go into such judgments, including the nature and degree of risk, the condition of the particular population involved, and the nature and level of the anticipated benefits.

Relevant risks and benefits must be thoroughly arrayed in documents and procedures used in the informed consent process.^19^

*Selection of subjects for research.* The third element, selection of subjects for research, finds its primary guidance in the principle of justice where the moral requirements demand that the procedures and outcomes for the selection of subjects are fair to the individual and within the social context. Participation in potentially beneficial research should be fairly distributed to all who wish to participate, and risky research should not be offered only to less desirable subjects. In the context of society, risks should be distributed after careful consideration of the burdens and the ability of individuals in identifiable groups to bear those burdens. As a generalization, adults should be considered before children, and participation by institutionalized individuals should invoke very careful consideration. Even with these safeguards, the National Commission believed that the selection of subjects may continue to reflect injustice arising from social, racial, sexual, and cultural biases institutionalized in society. Harking back to the ethical concerns that prompted the National Commission, the Belmont Report concludes with the following: “One special instance of injustice results from the involvement of vulnerable subjects. Certain groups, such as racial minorities, the economically disadvantaged, the very sick, and the institutionalized may continually be sought as research subjects, owing to their ready availability in settings where research is conducted. Given their dependent status and their frequently compromised capacity for free consent, they should be protected against the danger of being involved in research solely for administrative convenience, or because they are easy to manipulate as a result of their illness or socioeconomic condition.”^19^

Although not included in the body of the report, a footnote specifically addresses the difficulty in extrapolating these tenets to human subjects research in the social sciences: “Because the problems related to social experimentation may differ substantially from those of biomedical and behavioral research, the Commission specifically declines to make any policy determination regarding such research at this time. Rather, the Commission believes that the problem ought to be addressed by one of its successor bodies.”^19^ An appropriate ethical approach for some areas of social and psychological studies remains elusive. Matthew Salganik, professor of sociology at Princeton University, discusses the issues surrounding the difficulty in applying the Belmont Report recommendations at his blog.^20^

The Belmont Report was submitted to Congress on April 18, 1979.

### Other Reports by the National Commission for the Protection of Human Subjects in Biomedical and Behavioral Research

Although the Belmont Report is the centerpiece for the analysis of research in human subjects, the National Commission for the Protection of Human Subjects in Biomedical and Behavioral Research provided significant additional guidance for Congress to consider as the legislators moved forward to formulate regulations for the governance of human subjects in research. During the 4 years of the National Commission's appointment, other publications provided analysis of concerns related to specific questions (Table), and many of the recommendations were incorporated into the subsequent regulations for human subjects protection.^21^

**Table .** Human Subjects Protection in Research Reports From the National Commission for the Protection of Human Subjects of Biomedical and Behavioral Research, 1974-1978^21^

Report	Date
Research on the Fetus	July 25, 1975
Research Involving Prisoners	October 1, 1976
Psychosurgery	March 14, 1977
Disclosure of Research Information Under FOIA	April 8, 1977
Research Involving Children	September 6, 1977
Research Involving Those Institutionalized as Mentally Infirm	February 2, 1978
Institutional Review Boards	September 1, 1978
The Belmont Report	September 30, 1978
Delivery of Health Services	September 30, 1978
Special Study on Implications of Advances in Biomedical and Behavioral Research	September 30, 1978

FOIA, Freedom of Information Act.

### Principles of Biomedical Ethics

Another landmark publication from 1979 deserves attention for its sustained influence on the field of biomedical ethics and its deviation from the three ethical principles put forth by the Belmont Report. In *Principles of Biomedical Ethics*, Tom Beauchamp and James Childress argue for inclusion of nonmaleficence as an independent principle to formulate the now-familiar four principles that inform contemporary bioethical discourse.^22^ As previously noted, nonmaleficence is considered a duty under the umbrella of the principle of beneficence in the Belmont Report. Beauchamp and Childress maintained that the tradition to do no harm central to the tenets of the Hippocratic Oath incorporates the concept of nonmaleficence at its core and is essential to any discussion of the ethics of medical practice. As such, they argued, this concept should be considered as separate from and not subsidiary to beneficence: “First, to confuse them is to obscure distinctions that we make in ordinary moral discourse. Second, ordinary moral discourse expresses the defensible conviction that we have certain duties not to injure others that are not only distinct from but also more stringent than our duties to benefit others.”^22^ The authors make the distinction that the negative duty to cause no harm should be encompassed by nonmaleficence, and the positive but not so strongly established moral duty to benefit others should constitute the core of beneficence. The authors acknowledged that other eminent scholars disagreed with the separation of nonmaleficence and beneficence, but they constructed an argument that has been upheld by the historic inclusion of nonmaleficence in most bioethics discussions following their book's original publication (the book is now its seventh edition). The book has had a significant influence on the still-evolving field of bioethics contemporary to its publication and the Belmont Report. Both authors regularly served as staff members for the Kennedy Institute Intensive Bioethics Course, and Beauchamp served as staff philosopher for the National Commission for the Protection of Human Subjects in Biomedical and Behavioral Research that produced the Belmont Report. The authors acknowledged the influence of several other members of the commission and other colleagues who contributed significantly to their deliberations as their work progressed.

### US Legislative Updates, 1981

DHEW officially became the Department of Health and Human Services (HHS) in 1980, and in response to the Belmont Report, the HHS and the US Food and Drug Administration (FDA) significantly revised their protection of human subjects regulations in 1981 (45 CFR §46 and 21 CFR §50).^16,18,23^

These regulations specifically address concerns related to vulnerable populations in Subparts B, C, and D, incorporating the recommendations from the National Commission. The *Research on the Fetus* report^24^ informed Subpart B (additional protections for pregnant women, human fetuses, and neonates), Subpart C (additional protections for prisoners) reflected the recommendations in *Research Involving Prisoners*,^25^ and Subpart D (additional protections for children) was informed by the *Research Involving Children* report.^26^

## HUMAN SUBJECTS PROTECTION OVERSIGHT

### Oversight in the United States

To this point, this review has focused on some of the historic events and documents precipitating evaluation of the ethical requirements for human subjects research in the United States and a review of the regulations that evolved from that history. The question not yet addressed is how these regulations should be enforced. As with the discussion of research ethics, the approach to enforcement of regulations also lies within the National Research Service Award Act of 1974 (Pub L No. 93-348).^17^ In addition to establishing the National Commission responsible for the Belmont Report, the National Research Act elected to perpetuate the regulatory mechanism for research extant within many departments of DHEW that evolved from the US Public Health Service requirements initiated by the Surgeon General in 1966. The background of this development is described in William Curran's article, “Government Regulation of the Use of Human Subjects in Medical Research: The Approach of Two Federal Agencies.”^27^

This system for review of human subjects research within DHEW as described in *The Institutional Guide to DHEW Policy on Protection of Human Subjects*^28^ became the model for institutional review boards (IRBs) that the National Research Act would require of grantees and contractees for review of research involving human subjects. The National Commission for the Protection of Human Subjects in Biomedical and Behavioral Research was specifically charged with reviewing the function of IRBs and making recommendations for integrating the role of the IRB into the regulatory process to provide oversight of the application of ethical principles and of the regulations.^18^

On September 1, 1978, the National Commission completed the less spectacular but equally important report, *Institutional Review Boards*,^29^ before submitting the Belmont Report on September 30 that same year. *Institutional Review Boards* outlines the National Commission's concept of the ideal environment for the application of the federal regulations.

In the introduction to the report, the National Commission provides this understated assessment of the role of the IRB: “This review of proposed research by IRBs is the primary mechanism for assuring that the rights of human subjects are protected.”^29^ The document outlines the ideal responsibilities of the IRB in the oversight of research to ensure that human subjects receive appropriate protections and ethical treatment for their willingness to participate in research, sometimes at no benefit to themselves. The National Commission summarized their objective as follows:

In the recommendations that follow, the Commission expresses its judgment about the ways in which those elements [that must be considered in balancing society's interests in protecting the rights of the subjects and in developing knowledge that can benefit the subjects or society as a whole] ought to be brought to bear on research practices, so that a reasonable and ethical balance of society's interests may be attained.

The Commission's deliberations begin with the premise that investigators should not have sole responsibility for determining whether research involving human subjects fulfills ethical standards. Others, who are independent of the research, must share this responsibility, because investigators are always in positions of potential conflict by virtue of their concern with the pursuit of knowledge as well as the welfare of the human subjects of their research.

The Commission believes that the rights of subjects should be protected by local review committees operating pursuant to federal regulations and located in institutions where research involving human subjects is conducted.^29^

The document continues this proposal and is seemingly all-inclusive in its conception of the IRB. Highlights include a list of the requirements that must be met to approve research and details for reviewing and approving the consent process, including the essential elements to be included and the safeguards that should be in place to ensure that the process is respected. Specific recommendations also address the constitution of the IRB; how it should be funded; and legal protections for the board, the process, and its members.

Most of the recommendations from the *Institutional Review Boards* report were incorporated into the HHS regulations—HHS being the responsible federal agency—as part of the rules revision in 1981 in response to the Belmont Report and several other publications of the National Commission. Acting independently from HHS, the FDA also adopted IRBs as a regulatory mechanism, with regulations first issued in 1981 as part of the agency's response to provisions of the National Research Act.^18,30^

One particularly relevant recommendation of the National Commission from *Institutional Review Boards* remained outstanding after the changes in 1981: “Recommendation (1) (A) Federal law should be enacted or amended to authorize the Secretary of Health, Education, and Welfare to promulgate regulations governing ethical review of all research involving human subjects that is subject to federal regulation.”^29^

The report notes significant “variations arising out of differences in wording, imposition of additional requirements, introduction of minor changes, etc.” among the different agencies apart from DHEW involved in research involving human subjects and expresses concern that this variability places an unnecessary burden on the individual IRBs for interpreting and properly enforcing the regulations. The National Commission's recommendation was to establish “DHEW as the sole authority” for regulations, expressing the belief that such a rule “would reduce the burden on IRBs to interpret and apply the regulations to which they are subject. Moreover, uniformity would assure a minimum level of protection to human subjects of research, no matter which federal agency is supporting the research or which entity is conducting it.”^29^ Having inherited the mantle of responsibility from the now-extinct DHEW and recognizing the reality of this assessment, the newly designated HHS explored implementation of this recommendation, particularly as it related to the function of IRBs. As with most changes affecting multiple branches of government, the process became complex. In December 1981, the President's Commission for the Study of Ethical Problems in Medicine and in Biomedical and Behavioral Research, a new commission appointed by Congress in 1978, entered the fray and recommended that all federal departments and agencies adopt the HHS regulations (45 CFR §46). In addition, an ad hoc Committee for the Protection of Human Subjects—composed of representatives and ex officio members from departments and agencies that conducted, supported, or regulated research involving human subjects—was appointed in May 1982 by the president's science advisor to respond to the recommendations of this new commission. After much consideration and negotiation, these efforts were finally addressed by adoption of the Federal Policy for the Protection of Human Subjects, known as the Common Rule, in 1991 and codified in the individual regulations by 15 federal departments and agencies. Each of these agencies includes in its chapter of the Code of Federal Regulations (CFR) section numbers and language that are identical to those of the HHS codification at 45 CFR §46, Subpart A for the regulation of human subjects participation in research. The HHS regulations also include Subparts B, C, and D as additional regulations pertaining to vulnerable subjects.^16,31^

In addition to harmonizing the regulations across agencies of the federal government, the Common Rule requires institutions that receive funds for research involving human subjects from federal agencies that are signatories to the Common Rule to certify that the research has been reviewed and approved by an IRB that meets the specific requirements for composition, for functioning, and for the criteria followed to approve research. By mandate of the Common Rule, IRBs are empowered to approve, require modifications of, or disapprove research activities and are required to conduct continuing review of ongoing research at least annually.

The FDA concurs with the Common Rule but claims special privilege in not signing on to it. In the Federal Register of November 10, 1988 (53 FR 45678), the agency proposed to amend its regulations in 21 CFR §50 and §56 so that they conformed to the Federal Policy for the Protection of Human Subjects to the extent permitted but noted that the FDA is a regulatory agency that rarely supports or conducts research under its regulations.^32^

### International Oversight

With the adoption of the Common Rule, regulations for human subjects research conducted within the United States became well established, but research has never been confined by the borders of the United States. Even though a project funded by federal monetary support may have some leverage to require adherence to US regulations, significant numbers of human research subjects participate in studies well beyond the influence of the US regulations. The international norms for participation of human subjects in research evolved along a course that frequently cross-pollinated with the concepts culminating in the Common Rule. The Declaration of Helsinki was an early statement of basic tenets that should apply to all research involving human subjects, and it has continued to evolve, with updates reflecting new issues as they become relevant. While addressing the ethical concepts, the Declaration of Helsinki does not provide an organizational or regulatory framework for human subjects protection. Providing this framework on an international basis presented a challenge well beyond the challenge of harmonizing regulations across different federal agencies as was accomplished by the Common Rule. The difficulties encountered in implementing the Common Rule represent only a microcosm of the enormous task of harmonizing regulatory and organizational concepts across the borders of different cultures and political systems. However, this task was particularly relevant because of the evolution of research into an international enterprise with multicenter drug trials and the expansion of vaccine trials in children. Many of these studies are conducted by multinational contract research organizations that have access to populations of subjects with exposure to diseases that may not be widely encountered in the United States.

An argument can be made that the process for oversight of human subjects in research at the international level started in 1948 before the Declaration of Helsinki when the United Nations Educational, Scientific and Cultural Organization (UNESCO) joined with the World Health Organization (WHO) to establish a permanent Council for Coordination of International Medical Congresses, formally constituted in Brussels in 1949 as a nongovernmental organization with the purpose of facilitating “the exchange of views and scientific information in the medical sciences by securing continuity and coordination between international organizations of medical sciences, by making their work known, and by providing them with material aid where necessary.”^33^ The scope of activities gradually expanded to include collaborative efforts among international medical activities in addition to the coordination of participating congresses. In 1992, the name of the council was changed to the Council for International Organizations of Medical Sciences (CIOMS), and its statutes were revised to reflect the expanded role.^33^

The original council ventured into medical research by organizing a 1959 meeting in Vienna under the auspices of UNESCO and the WHO “to discuss the principles, organization and scope of ‘controlled clinical trials,’ which must be carried out if new methods or preparations used for the treatment of disease are to be accurately assessed clinically.” The executive secretary summarized the meeting: “The conference was in itself an experiment.”^34^

Following this meeting, the council became much more involved in considerations regarding research and particularly the participation of human subjects in research trials, eventually publishing *Proposed International Guidelines for Biomedical Research Involving Human Subjects* in 1982. The purpose of the guidelines was “to indicate how the ethical principles that should guide the conduct of biomedical research involving human subjects, as set forth in the Declaration of Helsinki, could be applied, particularly in developing countries, given their socioeconomic circumstances, laws and regulations, and executive and administrative arrangements.”^35^ This quote is from the background notes for *International Ethical Guidelines for Biomedical Research Involving Human Subjects* published in 1993 after discussion and reconsideration of the comments received in response to the proposed guidelines.^35^

The publication of the guidelines in 1993, soon after the name change to CIOMS, represented a landmark for international research ethics. The steering committee included an international staff of 24 members and an even larger list of advisors and consultants. The committee was co-chaired by Robert Levine from Yale University, who was listed as a “Special Consultant” on the Belmont Report and authored the first four articles for discussion in the appendix to the Belmont Report, and John H. Bryant, an American physician with a distinguished career in international medical practice. In addition to the Declaration of Helsinki, the *International Ethical Guidelines for Biomedical Research Involving Human Subjects* was strongly influenced by the Belmont Report as demonstrated by the inclusion of the following text under the heading General Ethical Principles:

All research involving human subjects should be conducted in accordance with three basic ethical principles, namely respect for persons, beneficence and justice. It is generally agreed that these principles, which in the abstract have equal moral force, guide the conscientious preparation of proposals for scientific studies.^35^

The guidelines acknowledge the evolution of the principles following the publication of the Belmont Report with the statement, “Beneficence further proscribes the deliberate infliction of harm on persons; this aspect of beneficence is sometimes expressed as a separate principle, non-maleficence (do no harm).”^35^

**Figure 3.**
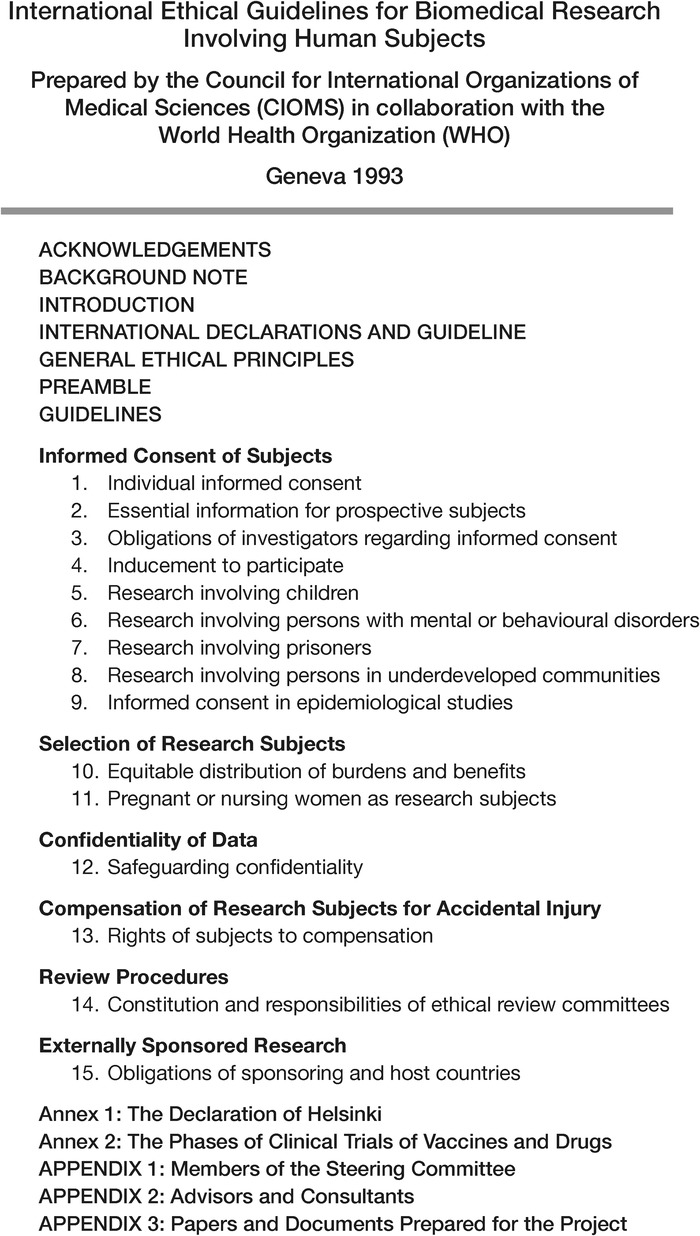
**Table of Contents, International Ethical Guidelines for Biomedical Research Involving Human Subjects, 1993^35^**

The table of contents of the 1993 *International Ethical Guidelines for Biomedical Research Involving Human Subjects*, provided in Figure 3, outlines the subjects the steering committee felt to be the most pertinent issues for research conducted in an international setting. In addition to the obvious influence of the Declaration of Helsinki, this document reinterprets many of the issues presented in the Belmont Report, in reports from the presidential commissions, and in 45 CFR §46, Subparts A, B, C, and D to provide an adaptable set of guidelines suitable for application across a broad spectrum of cultural and political environments. The notable exception to the similarities with the US regulations is the inclusion of a guideline titled “Compensation of Research Subjects for Accidental Injury” that provides for the following: “Research subjects who suffer physical injury as a result of their participation are entitled to such financial or other assistance as would compensate them equitably for any temporary or permanent impairment or disability. In the case of death, their dependents are entitled to material compensation. The right to compensation may not be waived.”^35^ To date, no uniform program for compensation of human subjects injured in research is addressed in the US regulations.

The *International Ethical Guidelines for Biomedical Research Involving Human Subjects* was updated in 2002, and CIOMS continues its efforts to revise the guidelines as dictated by changes in research requiring human subjects.

## THE GUATEMALA SEXUALLY TRANSMITTED DISEASES STUDY

All the efforts described to this point promoted regulations and procedures based on an ethically sound approach to protecting human subjects who, by consent or proxy, will be participating in research. The ethics of the research environment seems to have improved as a result of these efforts both in the United States and internationally. Notable instances of particularly egregious studies have come to light since the publication of the Belmont Report, but most of these studies originated before that document was issued. One study in particular raised eyebrows for its similarity to the transgressions committed in the Tuskegee study and, after investigation, was found to have ties to the Tuskegee study.

In October 2010, the United States disclosed that the US Public Health Service sponsored studies of sexually transmitted diseases in Guatemala beginning in 1946. This exposé began with the discovery of documents among papers donated by Dr John Cutler to the library at the University of Pittsburgh. Before retiring, Cutler was on the faculty at the university's School of Public Health following a long career in the US Public Health Service where he had been one of the staff members involved with the Tuskegee study. Hoping to gain insight into the Tuskegee study, Dr Susan Reverby from Wellesley was reviewing Cutler's papers when she came across previously unknown information about experiments investigating sexually transmitted diseases in Guatemala that Cutler and his associates conducted.^36^ An account in the *American Journal of Public Health* reports that “… more than 5000 uninformed and unconsenting Guatemalan people were intentionally infected with bacteria that cause sexually transmitted diseases” and many were never treated.^37^

**Figure 4.**
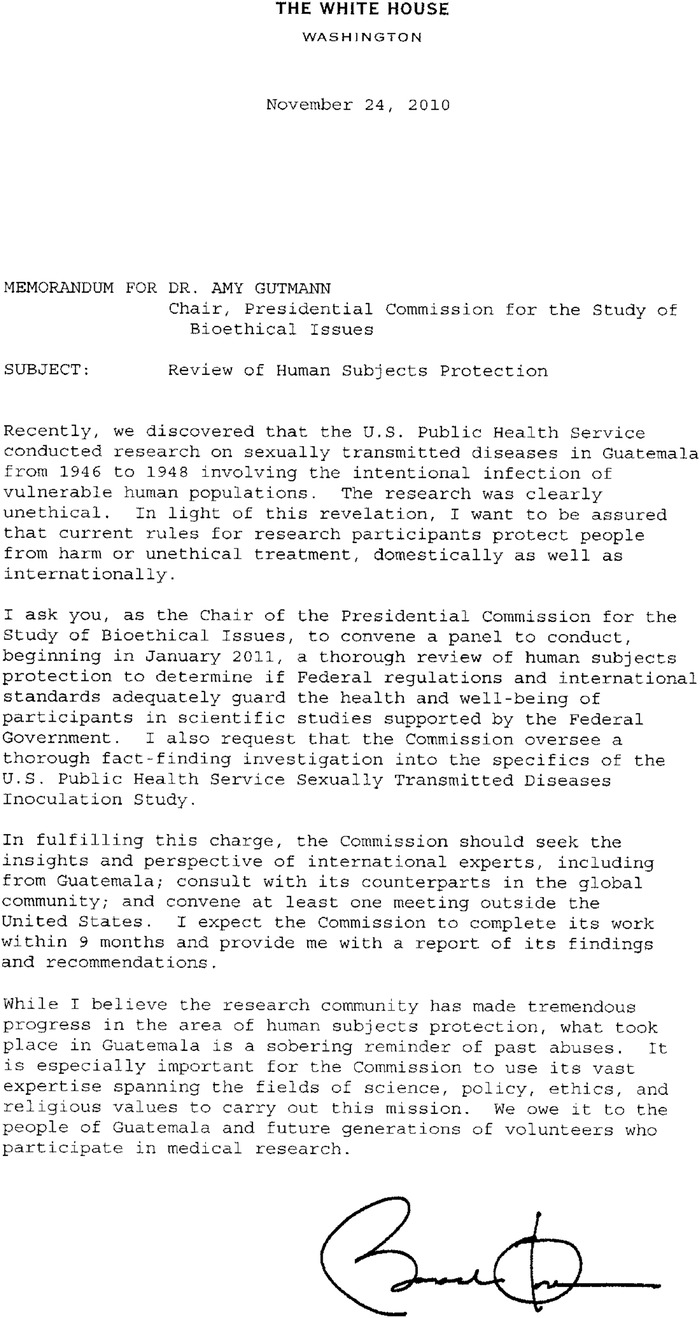
**Directive from President Barack Obama to investigate the Guatemalan studies, 2010^38^**

When the details of these experiments came to light, they precipitated an apology from President Barack Obama and specific directives to the Presidential Commission for the Study of Bioethical Issues, a commission appointed by Obama, to “convene a panel to conduct, beginning in January 2011, a thorough review of human subjects protection to determine if Federal regulations and international standards adequately guard the health and well-being of participants in scientific studies supported by the Federal Government. I also request that the Commission oversee a thorough fact-finding investigation into the specifics of the U.S. Public Health Service Sexually Transmitted Diseases Inoculation Study” (Figure 4).^38^

The Presidential Commission's first report, “*Ethically Impossible” STD Research in Guatemala from 1946 to 1948*, provides a detailed account of the history surrounding the Guatemala studies and all of the supporting evidence. In the preface, the Presidential Commission reports, “With dual responsibilities to give a full and fair accounting of events largely hidden from history for nearly 65 years and also provide an assessment of the current system, the Commission decided to publish two reports. This is the first report, a historical account and ethical assessment of the Guatemala experiments.”^38^

The specific political circumstances in which the experiments were conceived and carried out is critical to gaining some understanding of how ethically questionable research, however ill-conceived, was carried out by people who most probably had good intentions. The significance of the deleterious effects of sexually transmitted diseases among troops in World War II and how those effects precipitated the experiments are difficult to understand in today's world of effective antibiotics. In the 1940s wartime environment, however, understanding all aspects of sexually transmitted diseases was perceived as a crucial aspect of the military's ability to field an effective fighting force for the war in Europe. The experiments must be viewed in this historic context to understand the powerful motivation behind the studies.

The “*Ethically Impossible*” report includes an excerpt from a 1943 letter from Dr Joseph Earle Moore, Chair of the Subcommittee on Venereal Diseases under the National Research Council, to A. N. Richards, Chair of the Medical Research Committee of the Office of Scientific Research and Development, in which Moore wrote that he expected “approximately 350,000 fresh infections with gonorrhea [in the Armed Forces], [which] will account for 7,000,000 lost man days per year, the equivalent of putting out of action for a full year the entire strength of two full armored divisions or of ten aircraft carriers.”^38^ Moore estimated that the cost of treating the anticipated infections would be $34 million, equivalent to approximately $440 million today, adjusted for inflation.

Within this context, serious planning to meet the challenge of understanding and treating sexually transmitted diseases appears to have coalesced at the national level in 1942. Planning for these studies continued through the following year, with one of the principals suggesting “the possibility of using federal prisoners, Army prisoners, or conscientious objectors as an alternative” for research subjects.^38^ In 1943, experiments began at the US Penitentiary in Terre Haute, IN, that continued for 2 years. The focus of the experiments was on efforts to infect prisoners with *Neisseria gonorrhoeae* to test various methods for prophylaxis and treatment. Isolates of bacteria were applied directly to the penises of subjects in an effort to reliably infect the “volunteers.” However, the failure to reliably infect subjects in this fashion clearly indicated that studies of prophylactic techniques would not be possible with this approach, leading to consideration of other options.

The studies were performed under the direction of Dr John F. Mahoney, then head of the US Public Health Service/Venereal Disease Research Laboratory (VDRL) set up within the US Marine Hospital in Staten Island, NY. Mahoney directed the Terra Haute prison studies from his Staten Island laboratory, while 28-year-old Dr Cutler ran the studies at the prison. Following the end of World War II in 1945, the military support for the studies was less enthusiastic, but the Public Health Service remained committed to supporting the research with plans to move the research to Guatemala. A 1947 article Mahoney published in the *Journal of Venereal Disease Information* provides some insight into why the studies were moved: “It has been considered impractical to work out, under postwar conditions in the United States, the solution of certain phases concerned with the prevention and treatment of syphilis. These problems are largely concerned with the development of an effective prophylactic agent for both gonorrhea and syphilis and the prolonged observation of patients treated with penicillin for early syphilis. Because of the relatively fixed character of the population and because of the highly cooperative attitude of the officials, both civil and military, an experimental laboratory in Guatemala City has been established….”^39^

As fate would have it, a Guatemalan physician named Funes, who had served a fellowship at the VDRL and returned to Guatemala, was essential to the transition of the studies to his country. In August 1946, Cutler transitioned from Terra Haute to Guatemala at Funes's urging. Cutler staffed a clinic that provided the regular health inspections required for registered sex workers and suggested that the facility provide an environment of “normal exposure” through which sexually transmitted diseases could be more predictably transmitted. The studies in Guatemala evaluated possible prophylactic intervention “in cooperation with the Guatemalan Venereal Disease Control Department” that Funes directed and the local penitentiary “where exposure of volunteers to infected prostitutes would provide the testing opportunities.”^38^ Enrolling prisoners, a contained and restricted population, after they had had sexual intercourse with commercial sex workers known to be infected with sexually transmitted diseases, promised to establish, according to Cutler, a “rapid and unequivocal answer as to the value of various prophylactic techniques” through the preferred technique of “normal exposure.”^38^

After beginning with studies of “normal exposure” in prisoners, Cutler expanded the population of research subjects to include patients in a psychiatric hospital and again tried artificial means of infection, including scarification—mechanically damaging the skin and mucous membranes of the penis—to enhance the likelihood of infecting the subject. An even more aggressive study included at least 7 women in a psychiatric institution who were infected by the injection of syphilis specimens directly into the subarachnoid space surrounding the brain. Only 5 of them later received medical therapy.^38^ In addition, studies to follow the serology of children in a large orphanage were undertaken to better understand the specificity of tests for sexually transmitted diseases, an additional goal of the Guatemalan studies.

Studies in which subjects were intentionally infected were completed in the later months of 1948, and Cutler left Guatemala in December 1948 to join a WHO Disease Demonstration Team in India. From April 1949 to July 1950, this team worked to establish a venereal disease control demonstration in various parts of India and teach advanced methods of control for sexually transmitted diseases. Meanwhile, the US Public Health Service hired Funes and another Guatemalan physician, Dr Salvado, to continue “the observation of certain of the patient groups” after Cutler left Guatemala. Funes's staff collected data on residents of the orphanage, inmates of the penitentiary, individuals from the psychiatric hospital, schoolchildren, and the members of “various Indian tribes in the vicinity of Guatemala” who had participated in the experiments. Funes was hired to “advise concerning the clinical examinations of treated patients, their re-treatment as may be required, the collection of blood specimens for serologic examinations at periodic intervals, the preparation and shipment of all blood specimens collected for serologic examination” to the United States, and “the submission of such reports as may be necessary for the completion of the study of this patient group.”^38^ Based on the one report available in the Cutler Documents, Funes and his staff followed approximately 248 people from the mental institution, completing 243 blood draws and 170 lumbar punctures. Several of those subjects tested positive for syphilis during the follow-up experiments. The subjects from the psychiatric hospital were followed until at least 1953. The published work resulting from the Guatemala experiments also indicates that Funes continued to do serological testing on the children at the orphanage until at least 1949.

The experiments in Terra Haute were conducted and supported by many of the same people involved in the Guatemala experiments with the same goal of finding suitable prophylaxes for sexually transmitted diseases. However, throughout their discussion of the background leading to the experiments in the United States and the subsequent Guatemalan experiments, the Presidential Commission provides details of concerns voiced among those planning the studies. These details construct a compelling argument that all along the way there was an undercurrent of concern that the studies proposed were at the least controversial, most probably unethical, and in some instances arguably illegal. The Presidential Commission reached the conclusion that “Conducting the experiments in Guatemala provided an opportunity to work with reduced concern for some of the key obstacles associated with the Terre Haute experiments: fear of adverse legal consequences and bad publicity.” In a footnote to the report, the authors point out that “These concerns followed the researchers to Guatemala, however, as evidenced by some of their efforts to limit and restrict access to information about the work.”^38^

The Presidential Commission summarized their findings as follows: “In the Commission's view, the Guatemala experiments involved unconscionable violations of ethics, even as judged against the researchers’ own understanding of the practices and requirements of medical ethics of the day.” The report concludes

Although some individuals are more blameworthy than others, the blame for this episode cannot be said to fall solely on the shoulders of one or two individuals. The unconscionable events that unfolded in Guatemala in the years 1946 to 1948 also represented an institutional failure of the sort that modern requirements of transparency and accountability are designed to prevent. In the final analysis, institutions are comprised of individuals who, however flawed, are expected to exercise sound judgment in the pursuit of their institutional mission. This is all the more true and important when those individuals hold privileged and powerful roles as professionals and public officials. One lesson of the Guatemala experiments, never to take ethics for granted, let alone confuse ethical principles with burdensome obstacles to be overcome or evaded, is a sobering one for our own and all subsequent generations. We should be ever vigilant to ensure that such reprehensible exploitation of our fellow human beings is never repeated.^38^

The second charge from President Obama to the Presidential Commission was to provide a “thorough review of human subjects protection to determine if Federal regulations and international standards adequately guard the health and well-being of participants in scientific studies supported by the Federal Government.” The Presidential Commission addressed this directive in their report *Moral Science: Protecting Participants in Human Subjects Research* that was completed in December 2011.^40^

Regarding whether the regulations would prevent abuses similar to the studies in Guatemala, the Commission noted, “Existing evidence suggests both that the rules governing federal research today adequately guard against abuses analogous to those perpetrated in Guatemala in the 1940s and that current regulations generally appear to protect people from avoidable harm or unethical treatment, insofar as is feasible given limited resources, no matter where U.S.-supported research occurs.”^40^ The report summary continued as follows:

The current U.S. system provides substantial protections for the health, rights, and welfare of research subjects and, in general, serves to “protect people from harm or unethical treatment” when they volunteer to participate as subjects in scientific studies supported by the federal government. However, because of the currently limited ability of some governmental agencies to identify basic information about all of their human subjects research, the Commission cannot say that all federally funded research provides optimal protections against avoidable harms and unethical treatment. The Commission finds significant room for improvement in several areas where, for example, immediate changes can be made to increase accountability and thereby reduce the likelihood of harm or unethical treatment.^40^

The report outlines the Presidential Commission's observations and recommendations based on a thorough review of federally funded research, including studies that may involve human subjects in other countries. One issue the Commission raised was the general lack of accessibility to data: “there is no ready source that comprehensively describes its [the federally funded human research enterprise] basic characteristics, such as level of funding, or number of studies, subjects, or geographic locations. Instead, what exists are isolated pockets of information and some descriptive summaries.”^40^ This difficulty in acquiring information prompted the Presidential Commission's first recommendation to improve accountability through public access: “accountability can and should be refined through improving access to basic information about the scope and volume of human subjects research funded by the government.” The commission cites precedent for this recommendation from the Institute of Medicine–issued *Responsible Research: A Systems Approach to Protecting Research Participants*, with its recommendation to extend the oversight system to all research, regardless of funding source or research setting.^41^

Treatment and compensation for research-related injuries were also identified as an issue of concern, a subject that has been scrutinized regularly in past discussions as human research protection has evolved. Obama's Commission noted that this issue still required attention at the time of their review, pointing out that most other developed countries require sponsors, investigators, or others engaged in research to provide treatment or reimbursement free of charge to the subject for research-related injury or illness. As discussed earlier, one of the deviations from the general agreement between CIOMS and US regulations is the recommendation for subject compensation in the CIOMS guidelines. The Presidential Commission “draws a bright line affirming the view of most bioethicists and others, including the majority of nations supporting human subjects research around the globe, that human subjects should not individually bear the costs of care required to treat harms resulting directly from that research.”^40^ Recognizing that previous bioethics commissions and other advisory bodies had opined in favor of compensation or treatment for research-related injuries with relative silence by the government, the Commission advocated a response as to reasons for changing or maintaining the status quo. This issue remains open with no progress as this article is being written.

The Commission also asked that the OHRP examine, recognize, and define when protections delineated in foreign laws and regulations are accepted as equivalent to US regulations and exercise its longstanding authority to recognize these protections when available. Protections offered by international partners have been a source of confusion, as the federal regulations state that equivalent protections from international studies should be accommodated but do not provide guidance for how they should be defined. This directive has been reevaluated several times since its inception, including a specific request from the United Kingdom in 2007 to provide a determination of equivalence for human research protections afforded by UK regulations. As of the Commission's report in 2011, the OHRP had not formally recognized any country's protections as equivalent.

The Commission also noted that the FDA, while not signatory to the Common Rule, does adhere to the regulations at 45 CFR §46, Subpart A whenever possible and accepts data from foreign studies that comply with certain international standards for human subjects protection, such as studies that abide by good clinical practice, the Declaration of Helsinki, or certain host country regulations. This practice should provide a model to develop a system for recognizing equivalent protections as currently regulated by provisions in the Common Rule.

In its final recommendation, Promoting Current Federal Reform Efforts, the Presidential Commission called for broad reform of federal research rules and procedures beyond simply addressing equivalent protections.

The Commission supports the federal government's proposed reforms to:

a) Restructure research oversight to appropriately calibrate the level and intensity of the review activities with the level of risk to human subjects;

b) Eliminate continuing review for certain lower-risk studies and regularly update the list of research categories that may undergo expedited review;

c) Reduce unnecessary, duplicative, or redundant institutional review board review in multi-site studies. Regardless of the process used to review and approve studies, institutions should retain responsibility for ensuring that human subjects are protected at their location as protection of human subjects includes much more than institutional review board review. The use of a single institutional review board of record should be made the regulatory default unless institutions or investigators have sufficient justification to act otherwise;

d) Make available standardized consent form templates with clear language understandable to subjects;

e) Harmonize the Common Rule and existing regulations of the Food and Drug Administration, and require that all federal agencies conducting human subjects research adopt human subjects regulations that are consistent with the ethical requirements of the Common Rule; and

f) Work toward developing an interoperable or compatible data collection system for adverse event reporting across the federal government.^40^

Most of these provisions were included in the revisions to the Common Rule that updated the original provisions from 1991 and were effective January 21, 2019, with the exception of staged implementation of single IRB review for multisite studies. Twenty federal agencies follow the Common Rule, with the notable exception of the FDA. So far, no official indication of the FDA's intent has been provided, although the expectation is that some effort will be made to harmonize the regulations—at least in a similar fashion as previous agreements.

## OTHER HUMAN SUBJECTS PROTECTION FAILURES

This exposition of how we have arrived at the current rules and regulations for protecting human subjects who participate in research is lengthy but is at best an outline. Even this abbreviated history should elicit an appreciation of the complexity of the ethics surrounding protection of human subjects in research. A fair question is whether these provisions have significantly altered the landscape since Dr Beecher published his concerns in the *New England Journal of Medicine* in 1966. A cursory review turns up a few exceptions to the relative safety afforded by the current protections, with three that are particularly instructive.

### Jesse Gelsinger

Jesse Gelsinger had just turned 18, the legal age for consent, when he volunteered in 1999 for a phase 1 gene therapy study designed for treatment of ornithine transcarbamylase (OTC) deficiency. Phase 1 studies are designed primarily to determine the appropriate dose of a drug. Gelsinger was born with a mild form of OTC that was well controlled by diet and drug therapy; he had minimal risk of serious complications from the disease as long as he followed his treatment protocol. He did not stand to benefit significantly from his participation in the phase 1 study but felt that he should volunteer because of the knowledge that might benefit others.

Gelsinger died 4 days after receiving an experimental therapy consisting of a gene attached to an adenovirus that would theoretically serve as a delivery system to insert the new gene into the DNA of his liver cells. The death was unexpected in a relatively healthy 18-year-old, and the outcome precipitated a long and contentious investigation into how the protections that should have prevented Gelsinger from participating in the study were circumvented or ignored. The investigation uncovered questions regarding (1) information that should have been included in the consent form, (2) the actual risk posed by the study based on complications from similar studies that were not disclosed in reports to regulatory bodies, (3) why Gelsinger was enrolled in the study in violation of the protocol's inclusion/exclusion criteria, (4) the potential risk/benefit analysis based on the mild nature of his disease that would argue against his participation, and (5) an undisclosed conflict of interest for the director of the gene studies program that may have clouded decisions at critical points during conduct of the study.^42-45^

Examination of this study demonstrates that the protections afforded to subjects are well established but still depend on the assumption that the individuals responsible for every step of the evaluation and approval of studies and those who actually conduct the research all perform reliably in their roles.

### Johns Hopkins Lead Abatement Study

Another notable case revolves around the issues of appropriate consent, appropriate risks for children (or any vulnerable population), and disclosure of results obtained in research studies. The Kennedy Krieger Institute (KKI), an affiliate of Johns Hopkins Children's Center, conducted a study evaluating the effectiveness of lead abatement programs in low-income housing in Baltimore, MD during the 1990s. The study recruited families to live in houses either untouched or treated with different abatement techniques to determine which processes were most effective in protecting children from the significant neurologic effects of elevated lead levels that were endemic among children living in low-income housing in Baltimore. The goal was “to find a relatively inexpensive and effective method for reducing—though not eliminating—the amount of lead in children's homes and thereby reducing the devastating effect of lead exposure on children's brains.”^46^ A total of 108 families with young children were recruited to live in houses with lead levels ranging from none to levels just below the existing legal limit, and the children's serum lead levels were monitored. In two homes, the lead levels in the children crossed into toxic levels, but the families were not informed or advised to move out of the toxic environment. Eventually, a lawsuit was filed on behalf of the two children, and it raised significant ethical questions surrounding informed consent, appropriate risks, and disclosure of results that are reviewed at length in the article “With the Best Intentions: Lead Research and the Challenge to Public Health.”^46^ The Maryland Court of Appeals opinion equated the multiyear lead study with the Tuskegee study in its egregious disregard for research ethics in a vulnerable population.

### Ellen Roche

Ellen Roche was a healthy 24-year-old laboratory technician at the Johns Hopkins Asthma and Allergy Center. She volunteered to take part in a 2001 lung function physiology experiment in which normal pulmonary function in healthy volunteers would be manipulated by inhalation of hexamethonium, a compound that interferes with normal nervous system interaction with the lungs to mimic a mild asthma attack. Although it had been used in the 1950s to treat hypertension, hexamethonium fell into disuse as more effective drugs became available, and the FDA withdrew approval in 1972. Of note, hexamethonium was never approved as an inhaled medication. Roche was the fourth patient to receive hexamethonium in the trial. At least one previous subject had had mild persistent respiratory symptoms that the investigator dismissed as a cold. Roche became very ill, with significant pulmonary abnormalities presenting within 24 hours. The symptoms progressed to multisystem organ failure, and she died within a month.^47,48^

The ensuing investigation turned up several concerns:

1. The literature search relied on PubMed and one contemporary textbook of pulmonary medicine to explore the potential use of hexamethonium for the purpose proposed in the research plan. Neither source revealed any indication of concerns, although other databases and older textbooks warned of significant pulmonary complications associated with hexamethonium.
2. No request was made to determine if the FDA required an investigational new drug application, even though the medication was no longer approved and had never been approved as an inhalational drug.
3. The consent form referred to hexamethonium as a medication but failed to mention that FDA approval had been withdrawn.
4. A few subjects included in previous studies used inhaled hexamethonium with no mention of problems in the subsequent publications, but two subjects did have significant difficulties that were not reported as the investigator did not consider them related to the drug.
5. The hexamethonium used in the study was of chemical grade and was not prepared as a pharmaceutical agent.^47,48^

This list is not complete and raises many concerns, but the focus of the investigation became the lack of adequate research to confirm that the compound used to induce asthma symptoms was safe. The responsibility for this failure primarily attached to the investigator, with additional concern focusing on a review process that failed to follow proper procedures for approval of the protocol. During the follow-up, several articles from the 1950s reporting that hexamethonium could cause fatal lung inflammation similar to the pulmonary complications leading to the demise of Ellen Roche were identified. PubMed's coverage of the literature starts in the mid-1960s. In addition, review of the FDA records related to the withdrawal of hexamethonium in 1972 cited the drug's “substantial potential toxicity” as one element leading to the decision.^48^

## CONCLUSION

The examples of ethical issues from history and the scarcity of contemporary examples demonstrate that regulations for the protection of humans participating in research have evolved in a way that minimizes the probability of harm to subjects choosing to participate in research. These examples also reinforce the importance of individual responsibility to faithfully execute the requirements of their assigned roles. Failure of IRBs to provide appropriate review and oversight can lead to severe consequences, as can abrogation by the investigator to place the well-being of the subjects as the primary responsibility in any research protocol. Furthermore, these examples support the argument that no amount of regulation or oversight can completely remove the variable of individual failures to adhere to the rules or accept the responsibility associated with their role in research that may precipitate serious unexpected consequences. The rules and expectations for those charged with the review, administration, and performance of research requiring human subjects can only minimize the probability that these instances will occur. The point at which the primary responsibility of protecting human subjects from preventable harm deviates to focus on some other aspect of the research that leads to harm is rarely predictable. Simplified to the world of Monty Python, “Nobody expects the Spanish Inquisition!”

## Notes

This article meets the Accreditation Council for Graduate Medical Education and the American Board of Medical Specialties Maintenance of Certification competencies for Patient Care, Medical Knowledge, and Systems-Based Practice.
